# Social integration and mental health of Somali refugees in the Netherlands: the role of perceived discrimination

**DOI:** 10.1186/s12889-022-14655-y

**Published:** 2022-11-29

**Authors:** Emma Kuppens, Thijs van den Broek

**Affiliations:** Department of Socio-Medical Sciences, Erasmus School of Health Policy & Management, Erasmus University Rotterdam, PO Box 1738, 3000 DR Rotterdam, The Netherlands

**Keywords:** Mental health, Psychological distress, Integration, Refugees, Discrimination

## Abstract

**Background:**

We assess whether social integration is associated with mental health among Somali refugees in the Netherlands, and how this association is shaped by perceived discrimination.

**Methods:**

We performed linear regression and formal mediation analyses on Survey Integration Minorities data (*n =* 417) to assess whether the effects of two facets of social integration – Dutch language proficiency and informal contacts with natives – on mental health were mediated or suppressed by perceived discrimination.

**Results:**

Dutch language proficiency was positively associated with mental health, but also with perceived discrimination. Informal contact with natives was not significantly associated with mental health or perceived discrimination. There was marginally significant evidence (*p* < .1) that perceived discrimination suppressed the positive association between Dutch language proficiency and mental health.

**Discussion:**

Greater Dutch language proficiency appears to be beneficial for Somali refugees’ mental health, but this effect may partly be cancelled by the associated stronger experiences of discrimination.

**Supplementary Information:**

The online version contains supplementary material available at 10.1186/s12889-022-14655-y.

## Introduction

War and violence have since the 1980s led to a constant influx of Somali refugees in the Netherlands [1]. In 2022, more than 41,000 people of Somali origin were living in the Netherlands [2]. The traumatic events that many Somali refugees experienced [3] put them at increased risk of poor psychological health [4, 5]. Earlier research has highlighted pronounced within-group mental health differences among Somali refugees in various Western countries, for instance by employment status [6] and by the extent to which they identified with Somalia and/or the destination country [7]. The current study focuses specifically on social integration in explaining mental health differences within the group of Somali refugees in the Netherlands. It also explores the role of perceived discrimination in shaping the association between social integration and the mental health of Somali refugees.

Given that returning to the country of origin is often not a realistic or desired option for refugees, many tend to stay longer in the destination country [8]. Policymakers in the Netherlands grapple with how to shape the increasingly diverse Dutch society in a way that promotes mental wellbeing and inclusion of all its members, including refugees [9, 10]. The current study may inform this debate.

### Social integration and mental health

Social integration refers to “how migrants forge social relations which enhance their connectedness with the place in which they settle and the wider society around them” [11]. It is a multi-faceted concept [12], with key facets being proficiency in the language of the destination country and informal contacts with destination country natives without a migration background [13, 14]. As argued by Dagevos [12], social integration can be seen as related to, yet distinct from, cultural integration, i.e., the extent to which immigrants ascribe to the host society’s prevailing moral standards and values, and the extent to which they identify as members of the host society [14, 15]. Although the central focus of the current study is not on cultural integration, we will take aspects of cultural integration – specifically ethnic identification – into account when assessing the associations between facets of social integration and refugees’ mental health. We do so because social integration and cultural integration are closely intertwined, and because prior research suggests that the latter is associated with refugees’ mental health [7].

Social integration may benefit mental health in multiple ways. When social networks are diverse and include members of the native population of the destination country in addition to co-ethnics, this may open ways to important resources, e.g., information, socially distant from the refugee that could benefit their life chances, and potentially their health, in the destination country [16, 17]. In contrast, inadequate local social networks may constitute a barrier for refugees to explore services and opportunities potentially available to them [18].

Poor command of the language in the destination country hinders developing informal contacts and social relationships with destination country natives, and forms a direct barrier to access to community services [19–21]. Low language proficiency has also been linked to raised feelings of loneliness among Somali immigrants in Western countries [18, 22], and loneliness is, in turn, a known risk factor for depressive symptoms [23]. Moreover, communication difficulties arising from low language proficiency may complicate the contact with a mental healthcare professional, which may result in suboptimal quality of the care received [24]. This may be stressful and could, depending on the individual’s coping behavior, result in negative emotions, which, ultimately, is detrimental for mental health. Refugees may also refrain from seeking mental healthcare entirely due to fear that they cannot communicate adequately with healthcare professionals, or because they are embarrassed about their language abilities [25–27]. Low language proficiency may thus result in inadequate – or even absent – treatment of emerging mental health issues, which, in turn, can be expected to translate in suboptimal mental health [28].

### Discrimination

The premise of the current study is that perceived discrimination may partly shape the impact of social integration on mental health among Somali refugees. Somali refugees are a group with multiple minority statuses, and they may therefore be target of discrimination on multiple grounds (e.g., race/ethnicity or Islamic beliefs) [29]. The negative association between experiences of discrimination and mental health is well-established [30–34], also in Somali refugees [35–38]. Perceptions that society is discriminatory and everyday experiences of minor acts of unjust treatment because of one’s social identity are chronic stressors. More apparent personal discriminatory acts are moreover acute stressors that are superimposed on this chronic stress, and the combined stress resulting from discrimination is detrimental for mental health [39].

Experiences of discrimination may either be a catalyst or a nullifier of potential beneficial mental health effects of social integration. Assimilation theory [40] would lead one to expect that discrimination plays the former role. Social integration can reduce the social distance between ethnic minority and native majority members [41]. Informal contact and familiarity with ethnic minority members who have proficiency in the language of the destination country and orientate towards the destination society may make ethnic majority group members more accepting towards minority members. As a consequence, attenuation of prejudice and discrimination may be expected [17, 40, 42]. Moreover, socially integrated ethnic minority members might feel more recognized and develop positive attitudes towards the native majority group. Assimilation theory would thus lead one to hypothesize that the beneficial mental health effects of destination country language proficiency and informal contacts with destination country natives can partly be attributed to reduced experiences of discrimination.

In stark contrast with what assimilation theory would lead one to expect, a phenomenon coined the integration paradox has recently been noted by scholars focusing on the links between social integration and discrimination [43, 44]. The integration paradox postulates a positive, rather than negative, association between migrants’ aptitude to integrate and experiences of discrimination [43, 44], and it posits that the greater exposure to discrimination among more strongly integrated migrants may result in disengagement in and resentment towards the destination society [45].

There are various explanations for a positive association between social integration and perceived discrimination. Integrated ethnic minority members are more exposed to contact with native majority members in the public context than their counterparts who mainly surround themselves with members from their own ethnic group. This may increase the sense of intergroup competition and increase the desire to strongly support in-group interests. Moreover, discrimination is more easily detected by those who speak the language of the destination country well [43]. Integrated ethnic minorities in the Netherlands are also to a larger extent confronted with the negative public view towards and political debates about ethnic minorities [46], which may increase feelings of discrimination [43]. Warfa et al. [29] noted frustration among many Somali refugees with professional skills and a very good command of the English language in the United Kingdom and the United States about the lack of recognition for their skills in the destination countries. This frustration, the authors argued, put the refugees at increased risk of resentment and psychological distress [29]. Following this reasoning, it may be hypothesized that potential beneficial mental health effects of destination country language proficiency and informal contacts with destination country natives are, in part, cancelled out by stronger experiences of discrimination among those with better destination country language proficiency and more informal contacts with natives.

## Data and methods

### Sample

The current study draws on data from the controlled circulation file of the 2015 edition of the Survey Integration Minorities (SIM) (Dutch: *Survey Intergratie Minderheden*) [47, 48]. SIM is a repeated cross-sectional survey commissioned by the Dutch Ministry of Social Affairs and Employment and conducted by the Netherlands Institute for Social Research. It focuses on the structural and socio-cultural position of migrants in the Netherlands. The third round of SIM (SIM2015), collected between January and June 2015, was the first to include a subsample of migrants of Somali origin. Other groups interviewed were people with Turkish, Moroccan, Surinamese, Antillean, Polish and native Dutch backgrounds.

Andriessen and Kappelhof describe in detail how the Somali subsample was recruited [47]. Statistics Netherlands randomly selected 1747 individuals of Somali origin (i.e., born in Somalia or at least one parent born in Somalia) from the Dutch population registers. Of these persons, 10 could not be contacted, either because they were deceased or because they no longer had a valid address. The 1737 remaining persons were sent a survey invitation, and 626 of them participated in the survey (response rate: 36.0%). Of these respondents, 234 filled in the survey online and 392 participated in computer-assisted personal interviews (CAPI). The questionnaires for the face-to-face interviews and the web survey were identical, and both were available in Dutch as well as in Somali. Face-to-face data collection was done by 15 interviewers with a Somali background.

Respondents were informed about the goals of the research, and it was emphasized that information would be anonymized and that participation in the survey and consent were voluntary. In the controlled circulation file of the dataset, selected information (e.g., age) was recategorized in broader categories in order to render identification of individual respondents impossible. The Research Ethics Review Committee of the Erasmus School of Health Policy & Management at Erasmus University Rotterdam assessed and approved the secondary analysis of the controlled circulation file of the SIM2015 dataset on which we report here (Reference: 21-033).

Given our focus on refugees, we excluded second-generation migrants (*n =* 51) and migrants who migrated for other reasons than war, political reasons or religious persecution (*n =* 158). Given that information on formal refugee status was not available, we had to rely on self-reported migration reasons as a proxy measure. The exclusion procedure described here resulted in a reduction of the analytical sample to 417 respondents. Supplied analytical weights were used to account for selective non-participation in the survey.

### Measures

Mental health was measured with an alternative version of the Mental Component Summary of the 12-item Short Format Health Survey (SF-12) [49]. Respondents were asked how often in the last four weeks they “had a lot of energy”, “had difficulty to meet family or friends or plan other social activities due to physical health or emotional problems”, “felt calm and peaceful”, “felt downhearted and blue”, “participated less in activities or accomplished less than you would like as a result of any emotional problems”, and “were unable to do work or other regular daily activities as carefully as usual due to any emotional problems”. Response categories differed somewhat from the original SF-12: the four first-mentioned questions had four response categories ranging from (1) “all the time” to (4) “never”, and the final two questions had two response categories: (1) “yes” and (2) “no”. Where necessary, answers were recoded, so that higher values corresponded with better mental health. Following the procedure described by Schellingerhout [50], a summary scale ranging from 0 to 100 was subsequently derived.

Two facets of social integration were considered: Dutch language proficiency and the frequency of informal contact with Dutch natives. Three questions assessed whether the respondents experienced difficulties when having a conversation in Dutch, when writing in Dutch, and when reading Dutch newspapers, letters or brochures. The response categories for each of the three questions were (1) “often”, (2) “sometimes” and (3) “never”. For the first question, “not able to speak Dutch” (4) was presented as an additional response category. Responses were summed into a consistent scale (Cronbach’s α = .80) ranging from 3 to 10, with higher scores indicating better Dutch language proficiency.

With regard to informal contact with Dutch natives, respondents were asked how often native Dutch friends or neighbors visited them, how often they spent leisure time with Dutch natives, and how often they had contact with native Dutch friends or acquaintances. For the first two questions, response categories ranged from (0) “never” to (2) “often”. Response categories for the third question ranged from (0) “Once per month or less” to (2) “daily”. Summing the answers on the three questions to resulted in an internally consistent scale (Cronbach’s α = .73) ranging from 0 to 6, with higher scores indicating more informal contact with Dutch natives. Perceived personal discrimination was captured with the question: “Have you ever been discriminated by the Dutch population? If so, how often did this happen?”. Response categories ranged from “never” (1) to “very often” (5).

In the multivariate analyses we controlled for gender, age, partner status, parenthood, educational attainment, employment status, religiosity and identification with Somalia and the Netherlands. These variables were considered potential confounders, because they may be expected to be associated with social integration as well as with mental health [6, 7, 32, 37, 44, 51–54].

Four age categories were distinguished: 15-24 years old, 25-34 years old, 35-44 years old, and 45 years and older. With regard to educational attainment, we distinguished three categories: (1) primary education or less, (2) lower secondary education, (3) higher secondary or tertiary education. Partner status was measured with a dichotomous variable distinguishing between respondents with a partner living in the Netherlands and their counterparts without a partner or with a partner living in a different country than the Netherlands. A dichotomous variable was included to distinguish respondents with at least one child from their childless counterparts. Another dichotomous variable indicated whether or not respondents were in paid employment. Length of stay was calculated by deducting the year in which the respondent first migrated to the Netherlands from the interview year. Given the positively skewed distribution of length of stay in years, log transformation was subsequently performed. Two dichotomous measures were included to capture respondents’ identification with Somalia and the Netherlands. Both variables were coded as 1 for respondents who reported identifying themselves “strongly” or “very strongly” as Somali or Dutch, respectively, and as 0 if they reported identifying themselves as such only “somewhat”, “not”, or “not at all”. A final dichotomous variable was included to capture religiosity. Note that respondents in our sample almost exclusively (96.4%) reported being Muslim. We distinguished between respondents who attended religious services at least weekly and their counterparts who attended religious services less often. Sample characteristics are presented in Table 1.

**Table 1:** Descriptive statistics

	Mean / %	(SD)	Allowable range	Valid N
Mental health (alternative SF-12)	75.8	(18.1)	0-100	369
Dutch language proficiency	6.9	(2.0)	3-10	417
Informal contact w/ natives	2.9	(1.7)	0-6	385
Perceived discrimination	1.9	(1.1)	1-5	409
Female	49.6%			417
Age:				417
15-24	16.8%			
25-34	33.8%			
35-44	25.2%			
45 and older	24.2%			
Educational attainment:				401
Primary or less	42.4%			
Lower secondary	27.2%			
Higher secondary or tertiary	30.4%			
Partner in the Netherlands	33.8%			417
Has children	62.1%			417
Employed	22.1%			417
Length of stay in years	12.2	(7.7)	1-64	416
Identification w/ Somalia	87.8%			409
Identification w/ Netherlands	42.1%			406
Frequent attendance religious services	40.0%			405

*Data are from Survey Integration Minorities 2015; Data are not weighted; non-imputed data*

### Missing values

Information on one or more variables of interest was missing for 88 respondents (22.1%) in the analytical sample. Information on mental health (*n =* 48) and informal contact with natives (*n =* 32) was missing most often. Multiple imputation using chained equations was used to deal with missing data [55]. The underlying assumption is that information was missing at random (MAR), i.e. that differences between the distributions of missing values and the distributions of observed values could be explained by variables included in the imputation model [56]. The findings from the substantive analyses on 20 imputed data sets were combined into a single set of results following Rubin’s rules [57], which take the variability in results between the imputed datasets into account. An overview of the sample characteristics after multiple imputation and weighting is provided in Additional file 1: Appendix A.

### Analytical approach

We estimated a set of ordinary least squares regression models to predict mental health. We first regressed mental health on Dutch language proficiency, informal contacts with natives, and the range of control variables described above. In a second model we added perceived discrimination as an explanatory variable. We performed formal mediation analyses to assess whether the effects of Dutch language proficiency and informal contacts with natives on mental health were mediated (or suppressed) by perceived discrimination. Following the procedure proposed by Preacher and Hayes [58], bootstrapping was used to estimate the indirect effects of Dutch language proficiency and informal contacts with natives on mental health via perceived discrimination.

## Results

Results of the ordinary least squares regression models of mental health are shown in Table 2. Consistent with our expectations, the first model indicated that, after adjusting for the other variables in the model, Dutch language proficiency was positively associated with mental health. No significant association between informal contacts with natives and mental health was found. The model furthermore indicated that having a partner in the Netherlands, a strong identification with Somalia and frequent attendance of religious services were associated with better mental health.

**Table 2:** Results of linear regression models of mental health

	Model 1		Model 2	
	B	(SE)	B	(SE)
Dutch language proficiency	1.389*	(0.561)	1.571**	(0.561)
Informal contact w/ natives	−0.641	(0.550)	−0.577	(0.546)
Perceived discrimination			−2.393**	(0.866)
Female	2.279	(2.038)	2.331	(2.022)
Age:				
15-24	Ref.		Ref.	
25-34	1.498	(3.021)	1.008	(2.998)
35-44	0.677	(3.650)	−0.062	(3.627)
45 and older	−2.542	(3.948)	−3.482	(3.927)
Educational attainment:				
Primary or less	Ref.		Ref.	
Lower secondary	2.998	(2.304)	3.208	(2.279)
Higher secondary or tertiary	−1.302	(2.454)	−0.587	(2.447)
Partner in the Netherlands	4.552*	(2.054)	3.969†	(2.049)
Has children	−1.651	(2.299)	−1.696	(2.279)
Employed	0.864	(2.325)	1.087	(2.309)
Length of stay (log)	0.035	(1.700)	0.607	(1.696)
Identification w/ Somalia	6.707*	(2.717)	6.782*	(2.694)
Identification w/ Netherlands	−1.479	(1.815)	−1.755	(1.803)
Frequent attendance	6.139**	(1.989)	5.992**	(1.968)
Religious services			−2.393**	(0.866)
Constant	57.770***	(5.114)	60.181***	(5.148)
R^2^	.106		.124	

*Data are from Survey Integration Minorities 2015;* n = *417; Data are weighted; Multiple imputation using chained equations used to deal with missing data.* † p < .1, * p < .05, ** p < .01, *** p < .001

A key aim of the current study was to assess how the associations between facets of social integrations and mental health are shaped by perceived discrimination. Prior to adding perceived discrimination to our regression model of mental health, we regressed perceived discrimination on Dutch language proficiency, informal contacts with natives and the aforementioned range of control variables. The results of this analysis are presented in Additional file 1: Appendix B. After adjusting for the other variables in the model, a better command of Dutch was associated with higher perceived discrimination. This is consistent with the notion of the integration paradox, which posits that migrants with a stronger aptitude to integrate report more, rather than less, discrimination. No significant effect of informal contacts with natives on perceived discrimination was found.

The second mental health model, in which we included perceived discrimination as an additional explanatory variable, showed that stronger perceived discrimination was associated with worse mental health. After the addition of perceived discrimination to the model, the coefficient estimate of Dutch language proficiency became notably larger. Results of the formal mediation analysis with bootstrapped standard errors are presented in Table 3. The partial suppression of the beneficial health effect of Dutch language proficiency by perceived discrimination was borderline significant (*p* < .1). The coefficient estimate of informal contacts with Dutch natives did not change substantially after the addition of perceived discrimination to the model. We thus found no evidence of suppression of a mental health effect of informal contacts with Dutch via perceived exposure to discrimination.

**Table 3:** Results of mediation analysis

	Dutch language proficiency		Informal contact w/ Dutch natives	
	B	(SE)	B	(SE)
Reduced form estimate	1.389*	(0.561)	−0.641	(0.550)
Full model estimate	1.571**	(0.561)	−0.577	(0.546)
Indirect effect via perceived discrimination	−0.182†	(0.109)	−0.064	(0.090)

*Data are from Survey Integration Minorities 2015; n = 417; Data are weighted; Bootstrapped standard errors; Multiple imputation using chained equations used to deal with missing data.* † p < .1, * p < .05, ** p < .01

## Discussion

Drawing on Survey Integration Minorities data of 417 Somali refugees in the Netherlands we assessed whether social integration – operationalized as Dutch language proficiency and informal contacts with Dutch natives – was associated with mental health among Somali refugees in the Netherlands, and how this association was shaped by perceived discrimination. Our results suggest that, overall, greater Dutch language proficiency is beneficial for Somali refugees’ mental health. We did not find statistically significant evidence that informal contacts with Dutch natives impacted the mental health of Somali refugees. Results of the mediation analyses provided tentative evidence for the hypothesis derived from the notion of the integration paradox that the beneficial mental health effects of Dutch language proficiency are partly cancelled out by stronger experiences of discrimination among Somali refugees with a better command of Dutch.

Our finding that Dutch language proficiency was positively associated with refugees’ mental health is consistent with our expectations derived from prior work referred to in the introduction section. Taken together, this work suggested that poor command of the language in the destination country may give rise to feelings of loneliness and to inadequate or even absent treatment of emerging mental health issues. It is important to emphasize, however, that we could not test whether these potential mechanisms effectively underlay the positive association reported here between Dutch language proficiency and mental health.

The fact that we did not find any evidence of a positive association between informal contacts with Dutch natives and refugees’ mental health may be related to the way informal contacts with Dutch natives were operationalized. The emphasis in our measurement was on frequency of various types of contacts. However, quality of the contacts, e.g., the extent to which one can discuss intimate and personal matters, may be more important [59]. Research suggests that the quality of contact with neighbors tends to be lower in ethnically diverse neighborhoods [60], such as those where many Somali refugees live. People of Somali origin also tend to relocate relatively often [1]. As a consequence, the quality of the informal social contacts with Dutch natives of Somali refugees may often not be sufficient to meaningfully contribute to better mental health.

In addition to the beneficial effect of Dutch language proficiency, our results also highlight that refugees who strongly identified as Somali and refugees who attended religious services at least weekly had better mental health than their counterparts who did not identify strongly as Somali and who attended religious services less often. The desire to be part of a group is a likely driver of frequent attendance of religious services among Somali refugees in the Netherlands [1]. Recent research suggests that friendships with immigrant peers may be beneficial for immigrants’ mental health and psychosocial wellbeing [61, 62]. The positive mental health effects of social integration as well as of self-identification as Somali and frequent attendance of religious services reported in the current study may be attributed to the preference among immigrants noted by Phinney et al. [63] for retaining ties with the culture of origin while also forging ties to the society of settlement. Persistent ties with the culture of origin should not be perceived as a barrier to social integration [14, 64], and it is important that policymakers and practitioners recognize the importance, and possibly the complementarity, of both for refugees’ wellbeing.

In the current study, we perceived social integration and cultural integration as related yet separate concepts. This choice was arguably somewhat arbitrary, as these two types of integration have also been considered together as aspects of socio-cultural integration [14]. A broader focus on socio-cultural integration, rather than merely on social integration, would imply more elaboration on the mental health impact of values and identification with the destination country. Although we did not theorize about potential links between cultural integration and refugees’ mental health, we did include a key aspect of cultural integration in our models, namely self-identification with the Netherlands. We did not find evidence that self-identification with the Netherlands was associated with better mental health among Somali refugees.

Several limitations of the current study should be considered. Due to the cross-sectional design, causality cannot be inferred from the findings reported here. In pursuit of stronger evidence for causal links, future studies can adopt a longitudinal design and investigate, for example, the extent to which acquisition of greater Dutch language proficiency yields changes in perceived discrimination and/or mental health.

Secondly, the mental health measure included in the SIM dataset was based on the validated SF-12 Mental Component Summary [49], but, as described in the measures section, it had some notable differences, for instance with regard to the number of response categories on several items. Although used in various studies by the Netherlands Institute for Social Research [1, 50, 65], the alternative measure has thus far not been validated. The average mental health score reported here was rather high. Possibly, mental health problems were underreported due to the stigma on mental health issues that has been noted among Somali refugees [66–69]. Given the relatively low response rate, the high mental health scores may also reflect sample bias. We used the supplied analytical weights to correct for selective non-response, but these weights only took gender, age category, household size and municipality size into account [47]. If after these corrections people with good mental health were still more likely than their counterparts with suboptimal mental health to participate in the survey, the average mental health score reported here may be higher than in the overall population of Somali refugees in the Netherlands. We would like to emphasize, however, that the main focus of the current study was not on absolute levels of mental health, but rather on the impact of facets of social integration and perceived discrimination on the mental health of Somali refugees in the Netherlands.

In the light of the increasing diversity of Dutch society, it is crucial that mental wellbeing and inclusion of all its members is adequately promoted. Our results underscore the importance of understanding the challenges Somali refugees in the Netherlands are confronted with. There is a need for initiating and sustaining initiatives that encourage Somali refugees to learn the Dutch language and that provide them with tools to overcome language barriers to access services. In line with a recent police advice by the Netherlands Institute for Social Research [10], our results also suggest that such initiatives may particularly be successful in promoting the mental health of Somali refugees in the Netherlands when combined with actions that address discrimination.

## Supplementary Information

**Additional file 1.**

## Data Availability

The dataset analyzed during the current study is available in the DANS repository of the Royal Dutch Academy of Sciences and Arts, 10.17026/dans-xep-by9x.
