# Supplementary material for: Social integration and mental health of Somali refugees in the Netherlands: the role of perceived discrimination
Source: BMC Public Health. 2022 Nov 29;22:2223. doi: 10.1186/s12889-022-14655-y (PMC9710139; doi:10.1186/s12889-022-14655-y)
Supplement: Supplementary file 1 — Additional file 1. [file 12889_2022_14655_MOESM1_ESM.pdf]

Appendix A. Descriptive statistics after multiple imputation and weighting.

	Mean / %	(SD)	Allowable range
Mental health (alternative SF-12)	75.7	(18.0)	0-100
Dutch language proficiency	6.9	(2.0)	3-10
Informal contact w/ natives	2.9	(1.7)	0-6
Perceived discrimination	1.9	(1.1)	1-5
Female	45.0%		
Age:			
15-24	16.6%		
25-34	35.4%		
35-44	22.4%		
45 and older	25.5%		
Educational attainment			
Primary or less	40.5%		
Lower secondary	27.4%		
Higher secondary or tertiary	32.0%		
Partner in the Netherlands	35.2%		
Has children	59.5%		
Employed	22.9%		
Length of stay in years	12.3	(7.9)	1-64
Identification w/ Somalia	87.7%		
Identification w/ Netherlands	43.0%		
Frequent attendance religious services	42.1%		

Notes: Data are from Survey Integration Minorities 2015; n=417; Data are weighted; Multiple imputation using chained equations used to deal with missing data.

Appendix B. Results of linear regression model of perceived discrimination.

	B	(SE)
Dutch language proficiency	0.076*	(0.033)
Informal contact w/ natives	0.027	(0.031)
Female	0.021	(0.116)
Age:		
15-24	Ref.	
25-34	-0.204	(0.173)
35-44	-0.308	(0.211)
45 and older	-0.392†	(0.226)
Educational attainment		
Primary or less	Ref.	
Lower secondary	0.088	(0.131)
Higher secondary or tertiary	0.298*	(0.142)
Partner in the Netherlands	-0.244*	(0.119)
Has children	-0.019	(0.134)
Employed	0.093	(0.134)
Length of stay (log)	0.238*	(0.098)
Identification w/ Somalia	0.031	(0.156)
Identification w/ Netherlands	-0.115	(0.105)
Frequent attendance religious services	-0.063	(0.113)
Constant	1.008***	(0.294)
R^2	.135	

Notes: Data are from Survey Integration Minorities 2015; n=417; Data are weighted; Multiple imputation using chained equations used to deal with missing data.

† *p* < .1, * *p* < .05, ** *p* < .01, *** *p* < .001
